# *COL6A1* mutation leading to Bethlem myopathy with recurrent hematuria: a case report

**DOI:** 10.1186/s12883-019-1263-0

**Published:** 2019-02-26

**Authors:** Mengxin Bao, Fei Mao, Zhangning Zhao, Gaoting Ma, Guangjun Xu, Wenjuan Xu, Huan Chen, Meijia Zhu

**Affiliations:** 1 Department of Neurology, Affiliated Qianfoshan Hospital of Shandong University, NO.16766, Jingshi Road, Shandong, Jinan, 250014 CN China; 2 Department of Neurology, Liaocheng People’s Hospital, NO 67, West Dongchang Road, Shandong, Liaocheng City, 252000 CN China; 3 Department of Neurology, First People’s Hospital of Jinan, NO. 132, Daminghu Road, Shandong, Jinan, 250013 CN China

**Keywords:** Collagen VI, Bethlem myopathy, Hematuria, COL6A1, Collagen IV, Muscle dystrophy

## Abstract

**Background:**

Collagen VI-related myopathies are a spectrum of muscular diseases with features of muscle weakness and atrophy, multiple contractures of joints, distal hyperextensibility, severe respiratory dysfunction and cutaneous alterations, attributable to mutations in the COL6A1, COL6A2, and COL6A3 genes. However, no case of collagen VI mutations with hematuria has been reported. We report a 14-year-old boy who had both Bethlem myopathy and recurrent hematuria and who carried a known de novo COL6A1 missense mutation c.877G > A (p.G293R).

**Case presentation:**

The patient was a 14-year-old boy presenting with muscle weakness from 3 years of age without any family history. Six months before admission, he developed recurrent gross hematuria, three bouts in total, with the presence of blood clots in the urine. Next-generation sequencing of his whole-exome was performed. The result of sequencing revealed a de novo heterozygous G-to-A nucleotide substitution at position 877 in exon 10 of the COL6A1 gene. After treatment, the hematuria healed, but the muscle weakness failed to improve.

**Conclusions:**

Hematuria in Bethlem myopathy can be caused by COL6 mutations, which may be related to the aberrant connection between collagen VI and collagen IV.

**Electronic supplementary material:**

The online version of this article (10.1186/s12883-019-1263-0) contains supplementary material, which is available to authorized users.

## Background

Collagen VI-related myopathies are a spectrum of muscular diseases with features of muscle weakness and atrophy, multiple contractures of joints, distal hyperextensibility, and severe respiratory dysfunction and cutaneous alterations that are attributable to mutations in the *COL6A1*, *COL6A2,* and *COL6A3* genes, which encode collagen VI [1]. Bethlem myopathy is a relatively benign form of collagen VI-related myopathies [1, 2]. Type VI collagen is a crucial component of the extracellular matrix and is ubiquitous in many tissues, where it has close correlations with other collagens (e.g., collagen IV) [3]. No case of collagen VI mutations with hematuria has previously been reported. Herein, we describe a boy who had both Bethlem myopathy and recurrent hematuria and who carried a known de novo *COL6A1* missense mutation c.877G > A (p.G293R).

## Case presentation

The patient was a 14-year-old boy born of nonconsanguineous parentage presenting with muscle weakness from 3 years of age without any family history. He presented congenitally with decreased fetal movements and mild developmental motor delay with toe walking evident. He had normal mental growth. He was observed to have slowly progressive weakness of the proximal muscles of the extremities and the axial muscles of the trunk but was still able to perform activities of daily living without assistance. At the same time, it was difficult for him to climb stairs, jump, run, and rise from the floor, but he had no respiratory dysfunction. He had hyperkeratosis pilaris on the extensor surface of the legs and arms. Six months before admission, he developed recurrent gross hematuria, three bouts in total, with the presence of blood clots in the urine. There was no history of fever, lumbodynia, urinary tract infection, urinary frequency, trauma, edema, arthralgias, or skin rashes during the disease course.

On examination, respiratory and cardiovascular examinations were normal. There was follicular hyperkeratosis on the extensor surface. Tests of mental function and cranial nerves function were normal. His face, lip, tongue, and throat muscles were unaffected. His neck muscles were noticeably weak (Medical Research Council (MRC) grade 3/5). The muscle weakness in the limbs was symmetrical (MRC grade 4/5 proximally and 3–4/5 distally) with muscle atrophy of the shoulder girdle and lower legs. His sensations were undamaged, and muscle stretch reflexes were nonexistent. Neither joint contractures nor muscle contractions were apparent apart from contracture of the ankles and pes cavus.

Routine blood and stool tests were normal. Routine urine tests disclosed 3823 urinary red cells/μL and 16 red cell casts/μL. Proteinuria was 187.60 mg/day, and blood pressure and glomerular filtration rate were within the normal range. Urine red blood cell phase demonstrated that 80% of the urinary erythrocytes were abnormal. Cystoscopy was performed with no abnormal signs other than some blood clots. Abdominal ultrasonography and contrast-enhanced CT scan demonstrated no abnormal signs.

**Fig. 1:**
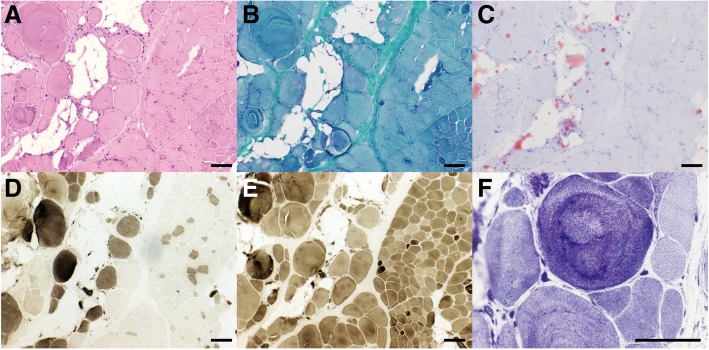
Muscular pathological findings (bar = 100 μm). **a** & **b**. H&E stain (**a**) and MGT stain (**b**): fibrosis, adipose tissue infiltration, rounding of muscle fibers, increased variability of fiber diameter, myonecrosis, and few regenerating fibers were seen. **c**. ORO stain: predominant adipose tissue infiltration was observed. **d** & **e**. ATPase stain(**d**: pH = 4.3, E: pH = 10.4): type-I and type-II fibers were affected equally with fiber type grouping. **f**. NADH stain: Disorganization of myofibril arrangement was noted on NADH stain

Electrocardiography and echocardiography were normal. Muscle enzymes were mildly elevated (CK: 394 U/L, 2-fold the upper limit of the reference range; CK-MB: 27.14 U/L, slightly higher than the upper limit). Nerve conduction tests of the patient were normal. Needle electromyography of the biceps brachii muscle revealed myopathic features with myotonic discharges and polyspike and polyphasic motor unit potentials (MUP) with early recruitment. The duration of polyphasic MUP was 8.9 ms, and the amplitude was 450.7uV. Muscle biopsy of the left biceps brachii in the patient revealed that the normal muscular structure was disturbed with fibrosis and adipose tissue infiltration. The muscle fibers varied in size. Small fibers appeared rounded in form, and hypertrophic myofibers could also be recognized. The numbers of central nuclei within the myofibers was increased (Fig. 1a, b & c). Muscle fiber necrosis with phagocytosis and regeneration presented in small groups. Type-I and type-II fibers were affected equally with fiber type grouping (Fig. 1d & e). Disorganization of myofibril arrangement was noted after NADH staining (Fig. 1f). Immunostaining with monoclonal antibodies against dystrophin (R, N, and C) revealed a normal staining pattern (not shown). We performed a collagen VI-stain by immunohistochemistry. The collagen-VI fibers were indistinguishable between the patient and the control (Additional file 1: Figure S1). Unfortunately, a renal biopsy was not performed due to a lack of parental approval. MRI of the thigh muscles revealed slight fat infiltration and bone marrow edema of the left collum femoris.

**Fig. 2:**
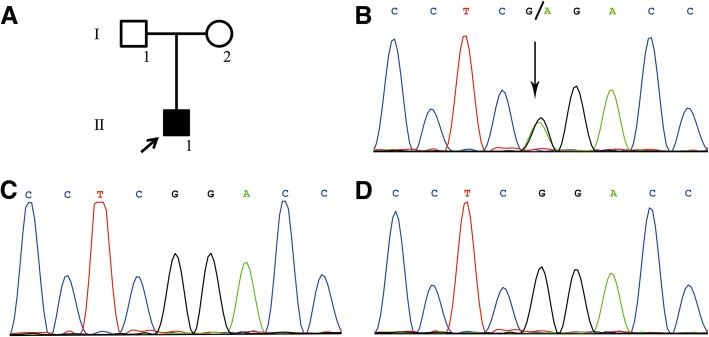
**a**. Arrow indicates the proband. **b**. Next-generation sequencing of whole-exome sequences revealed a de novo heterozygous G-to-A nucleotide substitution at position 877 in exon 10 of the *COL6A1* gene. **c** & **d**. The same mutation was not detected in his parents

Next-generation sequencing of the whole-exome was performed. The result of sequencing revealed a de novo heterozygous G-to-A nucleotide substitution at position 877 in exon 10 of the *COL6A1* gene (Fig. 2b), leading to an amino acid change of glycine to arginine, which had been previously described as pathogenic [4–6]. The same mutation was not detected in his parents. (Fig. 2c & d).

After treatment with idebenone 90 mg daily for 10 days, the hematuria healed, but the muscle weakness failed to improve.

## Discussion and conclusions

We report a 14-year-old boy who had both Bethlem myopathy and recurrent hematuria and who carried a known de novo COL6A1 missense mutation c.877G > A (p.G293R). He had developed features of Bethlem myopathy associated with this mutation, such as muscle wasting and weakness, joint contractures, pes cavus and follicular hyperkeratosis. Additionally, he had presented with recurrent gross hematuria. The findings of red cell casts and percentage of abnormal erythrocytes confirmed the glomerular origin of hematuria. There is no previous report on hematuria in Bethlem myopathy so far. Therefore, is there any association between the hematuria and the COL6A1 mutation? As we know, Bethlem myopathy is caused by mutations in the COL6A1, COL6A2, and COL6A3 genes. Collagen VI is ubiquitous in the extracellular matrix of many tissues, such as muscles, the nervous system, bone, cartilage, skin, tendons, adipose tissue, lung, kidney glomerulus, etc [7] Oomura et al. has confirmed collagen VI is strongly stained in the mesangium and interstitium and weakly stained along the glomerular basement membrane (GBM) by using a specific antibody in normal kidneys [8]. In extracellular matrix, collagen VI binds to cell-surface receptors and other extracellular matrix components (e.g., collagen IV). Previous studies colocalized collagen VI and collagen IV on the GBM using immunogold electron microscopy [9]. Huey-Ju Kuo et al. has demonstrated the amino-terminal domain of α1(VI) interacted with the carboxyl-terminal globular domain of type IV collagen [10]. The strong interaction of collagen VI with collagen IV provides a possible molecular pathogenesis for the hematuria of the case of Bethlem myopathy. Mutations in collagen IV genes may cause Alport syndrome, characterized by hematuria, renal failure, sensorineural deafness, and ocular abnormalities [11]. The missense mutation in COL6A1, c.877G > A, which is located in the conserved Gly–X–Y motif of the triple helical domain, altered the structure of the α1 chain and the protein conformation of collagen VI. Hence, we speculate that the connection between collagen VI and collagen IV is affected. Subsequently, the anchoring of GBM to the matrix is disturbed, which could lead to the GBM matrix being less highly cross-linked and more susceptible to proteolytic injury than normal GBM [12, 13]. Furthermore, a series of signaling pathways may be altered and result in complex cellular events. Unfortunately, due to his parents having rejected a renal biopsy, immunohistochemical analysis of renal tissue was not performed. Future studies will be needed to corroborate our suppositions.

## Additional file

Additional file 1: **Figure S1.** Collagen VI-stain by immunohistochemistry: The collagen-VI fibers were indistinguishable between the patient and the control. (TIF 11258 kb)

## Abbreviations

GBM: Glomerular basement membrane
MRC: Medical Research Council
